# CB2 Receptor in Microglia: The Guardian of Self-Control

**DOI:** 10.3390/ijms22010019

**Published:** 2020-12-22

**Authors:** Joanna Agnieszka Komorowska-Müller, Anne-Caroline Schmöle

**Affiliations:** 1 Institute for Molecular Psychiatry, Medical Faculty, University of Bonn, Venusberg-Campus 1, 53127 Bonn, Germany; jkimp@uni-bonn.de; 2 International Max Planck Research School for Brain and Behavior, University of Bonn, 53175 Bonn, Germany

**Keywords:** microglia, neuroinflammation, endocannabinoid system, cannabinoid receptor 2, neuron–microglia-communication

## Abstract

Microglia are key to maintaining the homeostasis of the brain. These immune cells of the brain can be our biggest ally in fighting infections, but can worsen pathology or hinder recovery when uncontrolled. Thus, understanding how microglia contribute to neuroinflammatory processes and how their activity can be controlled is of great importance. It is known that activation of endocannabinoid system, and especially the cannabinoid type 2 receptor (CB2R), decreases inflammation. Alongside its non-psychoactive effect, it makes the CB2R receptor a perfect target for treating diseases accompanied by neuroinflammation including neurodegenerative diseases. However, the exact mechanisms by which CB2R regulates microglial activity are not yet understood. Here, we review the current knowledge on the roles of microglial CB2R from in vitro and in vivo studies. We look into CB2R function under physiological and pathological conditions and focus on four different disease models representing chronic and acute inflammation. We highlight open questions and controversies and provide an update on the latest discoveries that were enabled by the development of novel technologies. Also, we discuss the recent findings on the role of microglia CB2R in cognition and its role in neuron–microglia communication.

## 1. Introduction

Microglia represent a powerful tool that our brain uses to maintain homeostasis. They not only take care of the debris and dead cells, but also actively fight against an assault. When the fight is over, they switch off their offensive behaviour and help in the healing process. Due to their destructive potential it is crucial to maintain control over their actions, but up to date this goal of keeping the balance is still elusive. Recent findings show that modulation of the endocannabinoid system (ECS), in particular the cannabinoid receptor 2 (CB2R), might be the key to the underlying control mechanism, as this receptor plays a main role in regulating the switch between the pro- and anti-inflammatory phenotype of microglia. This could be a crucial step in developing therapies to treat diseases accompanied by neuroinflammation including neurodegenerative diseases.

### 1.1. Microglia and Neuroinflammation

What is neuroinflammation? It is an inflammatory response in the brain. Various stimuli can induce it, including invading pathogens, tissue damage and aging. Neuroinflammation allows for a precise reaction to the assault and in later stages supports healing of the tissue. Unfortunately, besides activating the self-protection machinery, enhanced or prolonged neuroinflammation can result in irreversible damage that may lead to neurodegeneration or chronic pain. Therefore, keeping the balance between the positive and negative sides of neuroinflammation is of great importance.

The most critical mediators of neuroinflammation are microglia, the resident immune cells of the brain. They account for 10–15% of central nervous system (CNS) cells [1]. In contrast to other immune cells, they do not originate from myeloid hematopoietic stem cells, but develop from the yolk sac [2,3]. Whereas peripheral immune cells, such as macrophages or dendritic cells, are continuously replaced by myeloid progenitor cells, microglia cells can proliferate on demand and renew themselves, which promotes a rapid microglial response to threat.

Microglia constantly scan their surroundings by extending and retracting numerous protrusions and react to changes in their environment [4]. Under non-pathological conditions, they regulate growth and maintenance of dendritic spines [5,6,7]. Upon detecting neuronal damage or an assault, they become very motile, travel to the injury site to actively phagocytose debris and damaged neurons [8].

To efficiently detect encountered pathogens, microglia express a unique repertoire of receptors. These pattern recognition receptors (PRR) include FC receptors, viral receptors, anti-microbial peptides and toll-like receptors (TLR) [9] and recognize two types of patterns. They bind pathogen associated molecular patterns (PAMPs) expressed by microbes, and also recognize endogenous damage associated molecular patterns (DAMPs) [10].

As soon as microglia detect an infectious component, they rapidly change their morphology and trigger an inflammatory response, which include the phagocytosis of pathogens, the recruitment of other inflammatory cells and the production of inflammatory mediators such as different chemokines and cytokines [9]. The activation state of microglia in vitro is subdivided into three states: the homeostatic state (M0), the classical activation (M1) state or the alternative activation state (M2). Recent ideas proposed to rename the activation of a microglia/macrophages rather to their inflammatory profile dependent on the stimulus [11] or to their functional state (nurturer, sentinel, warrior) [9]. However, in this review, we will stay with the M1/M2 nomenclature, since it is used by the vast majority of cited publications.

M1 microglia aim to kill pathogens or infected cells. They no longer scan their surroundings, but retract their protrusions and increase their cell body size. This allows them to be more mobile and thus to actively migrate and phagocytose pathogens and cellular debris. Furthermore, they upregulate inducible nitric oxide synthase (iNOS), myeloperoxidase, and Nicotinamide adenine dinucleotide phosphate (NADPH) oxidase, as well as receptors that are associated with the adaptive immune response like major histocompatibility complex II (MHCII) or CD86. Extensive secretion of certain chemokines and cytokines such interleukin (IL)-1β causes a disruption of the blood brain barrier and enables the recruitment of peripheral macrophages via the CC-chemokine ligand (CCL) 2. This activation state can be induced in vitro by stimulation with lipopolysaccharide (LPS), interferon-γ (IFNγ), tumor necrosis factor (TNF)-α or other inflammatory mediators such as aggregated amyloid-β (Aβ), which is one of the key pathogenic hallmarks during Alzheimer’s Disease. In general, they induce the antigen presentation response to promote the adaptive immune system [12].

Once the threat has passed, the microglia switch to the M2 phenotype and try to dampen further inflammation and induce the healing process. They express anti-inflammatory mediators including different cytokines (IL-4, IL-10, IL-13), metabolic or tissue remodeling factors (chitinase-3-like protein 3 (YM-1), arginase 1 (Arg1)) or tropic factors (insulin-like growth factor 1 (IGF1), transforming growth factor-β (TGF-β)). They also facilitate phagocytosis of cell debris [13]. The M2 activation is subdivided into M2a, b and c, depending on the activator molecules. The M2a activation state is induced by IL-4 and IL-13 and is mainly involved in healing processes. The transitional activation M2b state is linked to immune regulation and driven by TLRs or IL-1R activation, whereas the acquired deactivated microglia M2c state is triggered by IL-10 and mainly involved in neuroprotection [14].

The different activation states of microglia with its positive and negative aspects are of great importance to keep an inflammatory balance in the brain. Within the last decade, several evidences arose that the ECS and especially CB2 can contribute to controlling the bidirectional activation of microglia.

### 1.2. The CB2 Receptor Is Expressed in the Brain not only under Pathological Conditions

The endocannabinoid system (ECS) is a neuromodulatory system that plays a major role in many vital processes from synaptic to immune modulation. It consists of endocannabinoids (ECs), their receptors, and synthesizing and degrading enzymes.

**Figure 1:**
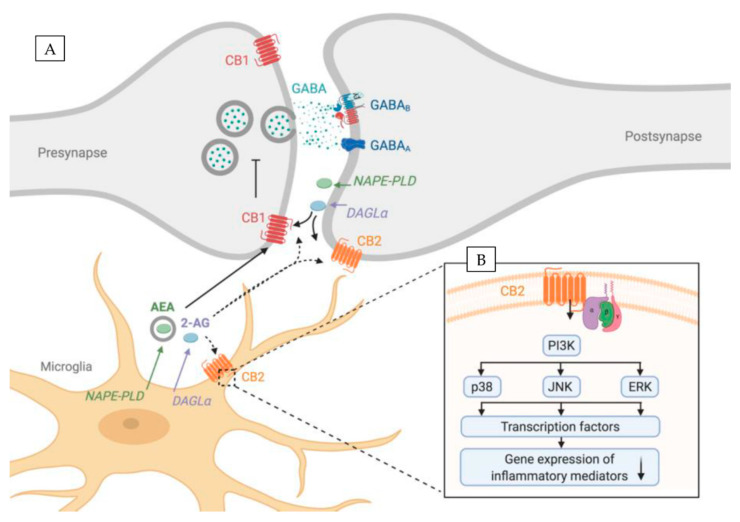
ECS components are expressed both by microglia and neurons. (**A**) Overview showing ECS components in both pre- and postsynaptic sites of neurons and in microglia. Full lines represent interactions supported by evidence, whereas dotted lines represent putative interactions. (**B**) Close-up showing downstream intracellular pathways of CB2R in microglia that regulate expression of inflammatory mediators. CB2R activation downregulates gene expression of inflammatory mediates (↓) via PI3K signaling. CB2R—cannabinoid receptor 2, CB1R—cannabinoid receptor 1, DAGL—diacylglycerol lipase-α, NAPE-PLD—N-acyl phosphatidylethanolamine phospholipase D, 2-AG—2-arachidonoylglycerol, AEA—arachidonoylethanolamide. Created with BioRender.com.

The most abundant ECs are small lipophilic molecules: 2-arachidonoylglycerol (2-AG) [15] and arachydonoylethanolamide (AEA, anandamide) [16] that are produced from membrane phospholipids in an enzymatic reaction. The main 2-AG synthesizing enzyme is diacylglycerol lipase-α (DAGLα), whereas for AEA it is N-acyl phosphatidylethanolamine phospholipase D (NAPE-PLD) (Figure 1A). Moreover, the exogenous cannabinoids such as cannabidiol or Δ9-tetrahydrocannabinol (THC) are well known and also have the ability to activate endocannabinoid receptors [17].

The two main cannabinoid receptors are the cannabinoid receptor 1 (CB1R, encoded by *Cnr1* gene) [18] and cannabinoid receptor 2 (CB2R, encoded by *Cnr2* gene) [19] (Figure 1A). Both cannabinoid receptors are G-protein-coupled receptors that consist of seven transmembrane domains, an extracellular N-terminus, and an intracellular C-terminus.

In the CNS, ECs are produced on demand, which involves the activation of Gq/11-coupled metabotropic glutamate receptors (mGluRs) or the mobilization of Ca^2+^ in postsynaptic spines. Subsequently, ECs are released into the synaptic cleft and in a retrograde fashion bind to the presynaptic CB1R (Figure 1A). This G-protein-coupled receptor is linked to Gi/o proteins, thus CB1R acts through the inhibition of the adenyl cyclase activity that in turn inhibits neurotransmitter release. This phenomenon is called depolarization-induced suppression of inhibition and depolarization-induced suppression of excitation, for inhibitory and excitatory synapses respectively. CB2R, on the other hand, is supposed to be localized on the postsynaptic neuron [20,21] (Figure 1A). Recent reports show that CB2R activation causes long-lasting hyperpolarization through the modulation of sodium-bicarbonate co-transporters. This self-inhibitory plasticity, mediated via 2-AG, was detected in the CA2/3 area of the hippocampus as well as in the somatosensory cortex [21,22]. Its modulation altered slow gamma oscillations in vivo [21] hinting its possible role in learning and memory [21]

CB1Rs are very prominently expressed in neurons, while CB2Rs were long believed to be mainly present on endocrine and immune cells and regarded as “peripheral cannabinoid receptor”. The initial analysis of *Cnr2* knockout (CB2^−/−^) mice showed a weak macrophage phenotype, but essentially normal responses to the CB agonist THC, which supported the idea that CB2 receptor plays a role rather in the periphery [23].

Although initial reports failed to reveal CB2R mRNA or protein in healthy brains [19,24,25,26,27,28,29,30,31], more recent studies suggest otherwise. Some reports investigating mRNA expression in healthy brain tissues from different rodent species and humans using in situ hybridization, Northern blot or RT-PCR methods confirmed the presence of CB2R also under physiological conditions [20,32,33,34,35,36]. Immunostaining and Western blotting of brain tissue from mice, rats, ferrets, and humans showed weak CB2R signals in several brain regions, including cortex, hippocampus, cerebellum, ventral tegmental area, and brain stem [32,33,35,37]. Moreover, studies in post-mortem human brains detected CB2R on perivascular microglia in the cerebellum [38].

These mixed findings indicate that CB2R are indeed expressed in the healthy brain, but at levels that are at the threshold of detection with most conventional methods. It was estimated that brain CB2R expression is approximately two orders of magnitude lower when compared to spleen tissue [20,34,35]. Characterization of CB2R in the brain has been a challenge not only due to its low baseline expression, but also due to the current lack of reliable antibodies [39,40,41].

While the neuroscience field debates the presence of CB2Rs in the healthy brain, many studies detected CB2Rs under pathological conditions such as neuroinflammation and neurodegeneration, which we discuss below. Evidence that macrophages upon inflammatory stimuli upregulate CB2Rs [42] brought to light that CB2R upregulation in the brain possibly arises from the activation of residing brain macrophages and immune cells of the brain—microglia.

Further evidence for CB2R expression in microglia came with recent technological advances. The development in the field of genetics enabled the creation of two reporter mouse lines in which CB2R expression is indicated by green fluorescence protein (GFP) expression [43,44]. In agreement with early reports in both reporter lines, high *Cnr2* expression was found in peripheral immune cells and the spleen, but CB2Rs were also detected in the brain. CB2-GFP BAC transgenic mice revealed microglial CB2R expression in healthy brain tissue [44], while CB2^EGFP/f/f^ showed no expression of CB2Rs under physiological conditions, but an upregulation in microglia in response to inflammation accompanying Alzheimer’s disease (AD) [43]. These different findings could potentially arise from different activation states of microglia in both experiments.

Nevertheless, more support for a CB2R expression in the healthy brain came with the use of novel techniques—RNAscope combined with immunohistochemistry and the combination of flow cytometry with qPCR [45]. Such studies showed that CB2Rs are expressed in glial cells as well as in excitatory and possibly inhibitory neurons in the hippocampus [21,36]. Further investigations showed neuronal CB2R expression in the midbrain dopaminergic neurons in the ventral tegmental area [35,46,47] as well as in the prefrontal cortex and cerebellum and lower levels in the dorsal striatum and nucleus accumbens [48].

As mentioned above, several reports suggest that the CB2R is expressed in the brain at low levels and is dynamically upregulated in response to neuroinflammation and other insults. This could be the reason why initially there were many conflicting reports regarding the presence of CB2R in the brain.

In summary, despite initial reports that CB2R is only expressed in the periphery it is now accepted that this receptor is present in both microglia and neurons in healthy brains and is upregulated by microglia during an assault. Here, we would like to focus on the role of CB2R in microglia under physiological and pathological conditions. Our review summarizes what is known so far and what are the open questions and controversies to be solved in order to use CB2R as a future therapeutical target.

## 2. The Role of CB2Rin Physiology and Neuroinflammation

What is the role of microglial CB2R? This question was extensively investigated in two different settings: in vitro and in vivo studies, mostly using mice as model organisms. Experiments done in vitro (summarized in Table 1) were usually performed with pharmacological tools to examine the underlying molecular mechanisms whereas in vivo studies (summarized in Table 2) also used knockout models to evaluate functional consequences of the CB2R deletion.

### 2.1. CB2R and Microglia In Vitro

CB2R activation in non-activated M0 microglia cells is closely linked to cell migration as CB2R activation of BV2 cells, an immortalized microglial cell line triggered cell migration [16,49]. This might be associated with CB2Rs expression in the leading edge of microglial lamellipodia [16]. This finding is important to understand how CB2R controls microglial function in terms of scanning their environment, but also in synaptic pruning.

Furthermore, it was demonstrated that CB2R mRNA is upregulated in microglia after stimulation with pro-inflammatory stimuli granulocyte-macrophage colony-stimulating factor (GM-CSF) and IFNγ [50]. However, LPS or LPS/IFNγ stimulation of cultured microglia cells decreased the expression of CB2Rs [50,51]. The observed differences could arise from the different nature of the compounds used for stimulation. IFNγ is a cytokine released during inflammation, whereas LPS is a bacterial toxin. Thus, it is likely that LPS causes a more severe inflammatory response that masks any specific effects mediated by IFNγ. Even though both LPS and IFNγ activate TLR4-receptors, their downstream signalling may differ in response to CB2R activation, as it has been shown that CB2R activates various signalling cascades (see Section 2.3). Further experiments are needed to examine the specific effects of CB2R stimulation on microglia activation using single treatments with either LPS or IFNγ.

Taken together, these early findings already suggested a potential role of CB2Rs during neuroinflammatory processes, which was further investigated and confirmed in several following studies.

**Figure 2:**
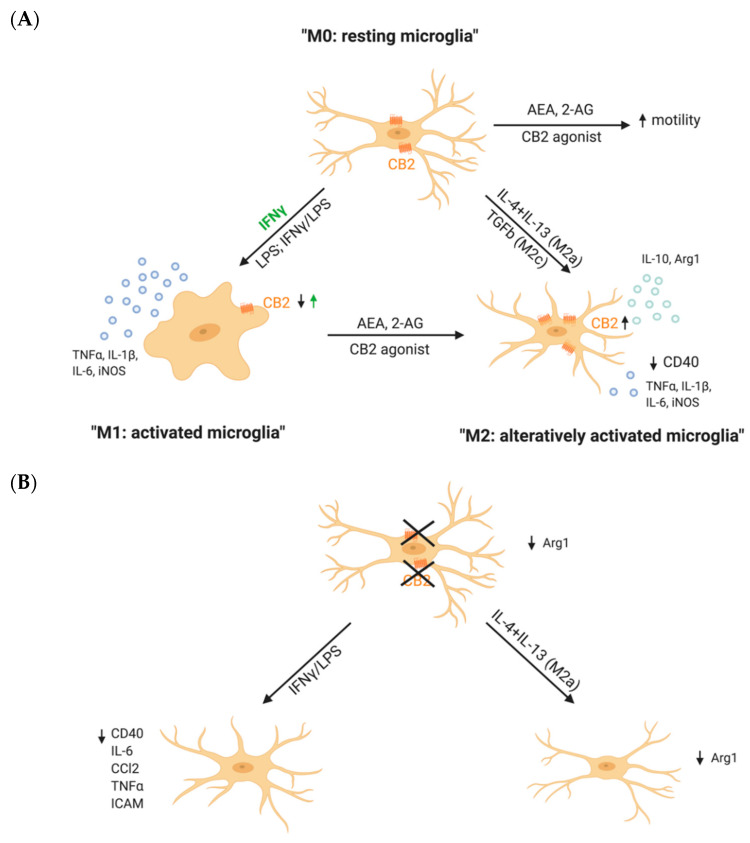
CB2R drives microglial polarization. (**A**) Results obtained from pharmacological studies in vitro showed that CB2R activation in unstimulated microglia (M0: resting microglia) results in increased microglial motility. Activation of microglia (M1: activated microglia) by IFNγ alone increases CB2R expression, whereas activation using a combination of IFNγ/LPS or LPS decreases CB2R expression and simultaneously increased the secretion of pro-inflammatory cytokines like TNFα, IL-1β and iNOS. Stimulation of CB2R with endo- and exogenous agonists causes a switch of microglia from pro- to anti-inflammatory states (M2: alternatively activated microglia). In this M2 state, microglia upregulate CB2R, decrease the secretion of pro-inflammatory cytokines and increase the expression of anti-inflammatory cytokines like IL-1β and Arg1. (**B**) Results from CB2^−/−^ in vitro studies suggest that the deletion of microglial CB2R leads to a suppression of inflammatory phenotypes. As IFNγ/LPS stimulation decreases the secretion of pro-inflammatory cytokines and expression of inflammatory markers. Similarly, alternative activation of CB2^−/−^ microglia using IL-4 + IL-13 does not take place, as Arg1 remains decreased. Small arrows represent the direction of the effects—an increase (↑) or a decrease (↓). The green arrow refers to an increase of CB2R after IFNγ stimulation. Created with BioRender.com.

The role of the CB2Rs in microglial activation can be investigated by either pharmacological or genetic approaches, so through pharmacological activation or blockage of the receptor or its genetic deletion. Pharmacological CB2R activation itself, under physiological conditions, did not alter the microglia activation state [52,53,54], whereas, in the presence of an inflammatory stimulus, activation of the CB2R by pharmacological compounds reduced IFNγ- or Aβ- induced release of pro-inflammatory mediators (Figure 2A) [52,55]. Similar observations were made in Aβ-stimulated rat microglia cultures. Here, TNFα levels were reduced after stimulation with different CB2R agonists [53]. Not only the secretion of pro-inflammatory cytokines, but also the phagocytic capacity of microglia was can be regulated by CB2R as CB2R activation diminished the CD40-mediated inhibition of Aβ phagocytosis [52].

One of the main ideas of how CB2R counteracts inflammation is through a shift in microglial polarization. Co-treatment of LPS/IFNγ-stimulated primary microglia with the CB2R ligand AEA increased the expression of IL-10, a marker for M2 polarization, in a dose-dependent manner [54]. This finding suggests that CB2R activation is able to diminish pro-inflammatory activation, but also to increase the expression of anti-inflammatory molecules. This was further supported by the finding that pharmacological activation of CB2Rs increased the expression of M2 markers by microglia, but decreased the expression of M1 inflammatory markers. In contrast, CB2R inhibition blocked this polarization effect [56]. Furthermore, CB2R expression was upregulated in both M2a and M2c microglia, but decreased in M1 microglia. In addition, CB2R activation of LPS/IFNγ-stimulated N9 microglia cells decreased the expression of the pro-inflammatory mediators, but increased the expression of anti-inflammatory mediators. This effect was reversed by treating the cells with the CB2R antagonist or an inhibitor of proteinkinase C [55].

Remarkably, microglia from CB2R deficient mice (CB2^−/−^) from *Cnr2*^tm1Dgen^ strain are not able to polarize to an M2a phenotype, as they showed a reduced expression of Arg1 after stimulation with IL-4/IL-13 (Figure 2B). Also, CB2^−/−^ microglia morphology was highly different from WT microglia morphology after inducing a M2a phenotype by stimulation with IL4 + IL13 [56]. However, it would be interesting to further investigate if microglia from CB2^−/−^ are able to differentiate to M2b or M2c subtypes, which is still unclear.

Interestingly, recent data from our lab showed that primary microglia from CB2^−/−^ from *Cnr2*^tim1Zim^ strain are less responsive to pro-inflammatory stimuli, which was also confirmed in a mouse model of AD [51]. One explanation would be that CB2R is crucial for microglia activation and its deletion impairs microglial activation to either pro- or anti-inflammatory phenotype.

However, this raises the question of potential differences between pharmacological and genetic models as well as differences between different genetic models itself to study the function of CB2Rs (see Section 2.4).

**Table 1:** Summary of CB2R-mediated effects on microglial activation in vitro. Outcome is either enhanced (↑), decreased (↓), altered (↔) or not altered (=).

Inflammatory Modulator	Model	CB2R Activity Modification	Outcome	Further Proof	Reference
Pharmacological modification of CB2R activity					
none	BV2 cells	ACPA	↑ Migration	CB1R antagonist did not affect ACPA-induced migration. CB2R antagonists inhibited migration	[49]
		2-AG	↑ Migration	cannabinol and cannabidiol blocks this effect	[16]
	Murine primary microglial cells	JWH-015	= NO, TNFα, CD40		[52]
	Rat primary microglial cells	HU-210; WIN 55,212-2; JWH-133	= TNFα		[53]
	Human/Rat microglial cells	AEA; 2-AG	↑ Arg1		[56]
LPS	Rat primary microglial cells	AEA; WIN 55,212-2	↓ iNOS,		
		AEA; 2-AG	↑ Arg1, SOCS3		
LPS/IFNγ	Murine N9 microglial cells	AM1241 (pretreatment)	↓ iNOS, ↑ Arg1, IL10, BDNF, GDNF ↓ TNFα, IL1β, IL-6	Effects reversed with CB2R antagonist AM630 or an inhibitor of protein kinase C	[55]
	Murine primary microglial cells	AEA; JWH-133	↑ IL-10	Effect reversed by CB2R antagonist SR144528	[54]
IFNγ		JWH-015	↓ CD40		[52]
IFNγ/CD40L Aβ_1–42_/CD40L		JWH-015	↓ NO, TNFα ↑ phagocytosis (of Aβ)		
Aβ	Rat primary microglial cells	HU-210; WIN 55,212-2; JWH-133	↓ TNFα ↓ microglia activation ↔ microglia morphology		[53]
IL-4/IL-13		none	↑ 2-AG, Arg1	Arg1 increase blocked by CB1R and CB2R antagonists	[56]
TGFβ		none	↑ AEA		
**Genetic deletion of CB2R**					
none	Murine primary microglial cells	*Cnr2* ^tm1Dgen^	↓ phagocytosis ↓ Arg1		[56]
LPS/IFNγ		*Cnr2* ^tim1Zim^	↓ TNFα, ICAM, CD40, IL-6, CCL2 = phagocytosis		[51]
IL-4/IL-13		*Cnr2* ^tm1Dgen^	↓ Arg1 ↔ microglia morphology ↓ phagocytosis		[56]

Altogether, in vitro studies demonstrate an important role of the CB2R on microglial activity by shifting the microglial phenotype from an inflammatory M1 activation rather to an anti-inflammatory M2 activation state (Figure 1). However, detailed studies on how CB2R activation affects microglial function, such as motility or phagocytic capacity, depending on their activation state M1 or M2 are still missing.

### 2.2. CB2Rs and Microglia: In Vivo Studies

CB2Rs are upregulated on microglia in the context of several neuroinflammatory diseases [57].

Since the summarized data from in vitro studies suggest that CB2Rs activity can alter microglia polarization, we aim to discuss these results also in the context of neuroinflammatory diseases. Here, we will discuss the role of CB2Rs in four different disease models that represent chronic inflammation in the central and peripheral nervous system, a genetic-based disorder and acute inflammation.

#### 2.2.1. Alzheimer’s Disease

Alzheimer’s disease (AD) is a neurodegenerative disease, which makes 60% of the dementia forms among the elderly population. Due to the increase in human life expectancy and the increased AD prevalence with age, the disease gains more and more scientific interest. AD is mainly characterized by pathological hallmarks such as brain atrophy, amyloid-β plaques and neurofibrillary tangles, which are accompanied by chronic inflammation and cognitive impairment.

AD is associated with an aggregation of miscleaved extracellular Aβ plaques and intracellular neurofibrillary tangles of phosphorylated tau. These accumulations greatly disrupt neuronal functions and can ultimately lead to neuronal death. Microglial cells detect the problem, get activated and are capable of aiding neurons by phagocyting Aβ plaques. Alternatively, they form a protective barrier around Aβ plaques, shielding them from the surrounding and hence ameliorate plaque-mediated neurotoxicity [58]. Furthermore, microglia can also aid neurons by phagocytosing extracellular tau tangles. This process is impaired during later stages of AD and the increased levels of tau lead to enhanced activation of microglia [59].

Besides phagocytosis, microglia respond to the presence of Aβ plaques with enhanced secretion of pro-inflammatory chemokines and cytokines, nitric oxide and free radicals. The release of chemokines and cytokines recruits myeloid cells from the periphery capable of phagocytosis, but it can also further enhance neuroinflammation. The increased pro-inflammatory signal results in over-activation of microglia leading to uncontrolled responses that subsequently result in neuronal dysfunction and cell death [60].

Enhanced CB2R expression was found on plaque-associated microglia from human AD brain tissue [61] as well as in different AD mouse models [62,63]. Beside this, an increase in the expression of CB2Rs in human brain tissue was detected, which was accompanied by enhanced levels of Aβ level and increased severity of neuritic plaque [64].

The influence of pharmacological components affecting CB2R activity in the context of AD-dependent neuroinflammation has been studied in various models consisting of in vitro studies by treating microglia cells with Aβ peptides. In vivo studies included at least three different genetic mouse models for AD (amyloid precursor protein (APP)-dependent, Presenilin (PS)-dependent or Tau-dependent). Whereas APP- and Presenilin- dependent mutations cause a Aβ-plaque-dependent pathology, mutations of Tau cause an AD-pathology that is based on neurofibrillary tangles. Both models reflect the neuropathological hallmarks of AD, but also have limitations, as they never represent the complete pathology. Since the AD pathology is age-dependent, the timing of treatment and analysis is of great importance. Studies are either performed before or at the beginning of the onset of pathology (early or pre-symptomatic) or at the late stage, where the pathology with Aβ plaque burden and neuroinflammation is fully developed and already results in cognitive impairment. However, most of the studies investigate the overall effect of CB2R activation on AD pathology rather than the specific role in microglial activation.

As microglia play an important role in AD pathology, controlling the microglia function would be a key treatment strategy. An upregulation of CB2Rs in microglia from AD patients raises the question if manipulation of CB2R activity can aid in controlling microglial activation [61]. CB2R activation in the early and pre-symptomatic phase in APP/PS1 mice decreased AD-dependent neuroinflammation. The treatment reduced the secretion of pro-inflammatory cytokines, but did not affect the Aβ plaque load. However, it improved the cognitive performance of mice. Interestingly, this effect was more pronounced when the treatment was performed during the early-symptomatic phase than during the pre-symptomatic state [65]. In agreement with that, it has been shown that chronic administration of the synthetic cannabinoid agonist did not improve memory performance of pre-symptomatic mice [66]. Furthermore, long-term treatment of early-symptomatic mice with the phytocannabinoid cannabidiol prevented cognitive deficits, as evaluated with the social recognition test, but did not affect the plaque load [67]. Beneficial effects of cannabidiol on AD pathology would be of interest for therapeutic aspects, as cannabidiol does not cause psychotropic effects like THC [68], but this needs to be further investigated.

Taken together, this suggests that CB2R plays a role rather in modulating the symptoms than in pathogenesis of the disease. Moreover, these findings support the idea that reducing neuroinflammation may be more relevant to cognitive performance than reducing plaque load. Similar observations were made after chronic oral CB2R agonist administration reduced the number of microglial cells as well as TNFα levels, suggesting that CB2R activation diminishes AD-dependent microglial activation. Here also cognitive performance was improved during the early-symptomatic phase. In contrast to the previous study, the memory improvement was associated with reduced plaque load [69]. Furthermore, beneficial effects of CB2R activation on AD-dependent neuroinflammation were also observed in late-symptomatic mice. Here, a long-term treatment with CB2R agonist decreased microgliosis and restored dendritic complexity in the cortex, but not in the hippocampus. This was reflected by improved performance in the cortex-dependent novel object recognition, whereas the performance in the hippocampus-dependent spatial memory Morris Water Maze (MWM) task was not altered [70]. These data suggest that the effect of CB2R activation on neuroinflammatory processes might also differ between brain regions.

In summary, most studies propose a beneficial effect of CB2R activation on AD pathology, which is most likely linked to reduced neuroinflammation. It seems that the timing of CB2R activation during AD pathology (pre-symptomatic phase versus early symptomatic phase versus late-symptomatic phase) plays an important role. Further studies should include a time-dependent expression profile of CB2Rs to provide more insights on the role of CB2Rs on AD-mediated microglial activation at different disease stages.

Another understanding in the mechanism of how CB2R activity controls microglia during AD-dependent neuroinflammation is provided by the genetic deletion of CB2Rs in AD mouse models. Aso et al. [71] investigated the role of CB2R deletion in pre-/early-symptomatic APP/PS1 mice on cognitive performance, microgliosis and plaque load [71]. They did not observe any effect of CB2R deletion, which is most likely due to the age of the tested animals as three and six months is still during the early phase of AD pathology. At that age the plaque formation already started but mice do not show any symptoms of cognitive impairment. Moreover, studies performed with late-symptomatic mice after CB2R deletion show contradicting results. Koppel et al. reported an enhanced number of plaque-associated microglia in late-symptomatic mice after CB2R deletion accompanied by enhanced levels of soluble Aβ_42_ levels and Aβ plaques [72]. In contrast, our study on late-symptomatic mice showed decreased levels of Aβ plaques. We focused on the inflammatory phenotype and how it influences neuronal loss and cognitive performance [51,73]. Deletion of the CB2R led to decreased microgliosis and reduced number of infiltrating cells from the periphery, which was subsequently accompanied by decreased neuronal loss and improved cognitive performance [51,73]. Moreover, we plaque-associated microglia in the knockout mice were more ramified, which indicated that they were less activated. This strongly supports the idea that the CB2R is able to influence microglial activity in AD-dependent neuroinflammation. Nevertheless, the difference between the results may arise from the fact that two different CB2R knockout lines were used for the analysis of the late-symptomatic mice (see Section 2.4).

Interestingly, the results between pharmacological and genetic manipulation of the CB2R in AD mouse model differ regarding their effect on microglial activity and AD-induced neuroinflammation. Pharmacological activation of CB2Rs reduced the secretion of pro-inflammatory cytokines and improved cognitive behaviour [65], but also deletion of the CB2R caused similar effects [51,73]. This difference can be due to the experimental design: pharmacological stimulation was used when mice were fully developed, whereas the knockout of CB2Rs happened already prenatally and continued on throughout mice life. This arises the question whether CB2Rs have a different effect at different stages of the disease. This question can be addressed in the future by using conditional knockout mouse lines (see Section 2.4).

However, the deletion of CB2Rs could have a more preventive effect, whereas acute activation of CB2Rs could lead to more curative effects. To answer this question, we need further studies looking into intracellular signalling of CB2Rs as well as its role in the immune response.

#### 2.2.2. Huntington’s Disease (HD) 

Huntington’s disease is a neurodegenerative disorder caused by multiple CAG trinucleotide repeats in the mutated *HTT* gene, which encodes huntingtin protein. HD is mainly characterized by motor impairment, mental disorders and cognitive deterioration. Beside neuronal damage [74], neuroinflammation and microgliosis are one of the main pathological features of HD [75]. Inflammatory markers such as IL-1β, IL-6, IL-8, and TNFα were detected in the plasma as well as in the cerebral fluid of HD patients [76,77,78]. HD animal models are created either by introducing the mentioned genetic mutation (R6/2 or BACHD mouse lines) or by pharmacological induction. For the latter, the so so-called malonate model is based on an intra-striatal injection of the mitochondrial complex II inhibitor malonate. The injection of malonate reflects the mitochondrial defects, which are found in HD patients [79].

The ECS is affected during HD, as the expression of CB1Rs is strongly downregulated in medium spiny projection neurons of the caudate and putamen [80,81,82]. Silencing and deletion of CB1Rs aggravated cognitive, behavioral, and motor deficits of HD pathology in different animal models [83,84,85]. Besides CB1Rs, CB2R activity has also been shown to contribute to the HD pathology.

CB2R expression in the brain was increased in tissue from both the genetic and malonate HD mouse models [86,87,88], but not in human tissue [89]. However, genetic deletion of CB2Rs in HD mice intensified motor deficits and decreased the life span [86,87]. This was accompanied by an enhanced microglia activation [87]. Furthermore, activation of CB2Rs extended the lifespan, improved motor functions and decreased CNS neuroinflammation. This effect was blocked by a CB2R antagonist specific to peripheral immune cells. In summary, these data stress the importance of peripheral immune cells and CB2Rs in the pathology of HD [86].

#### 2.2.3. Neuropathic Pain

Neuropathic pain appears after nerve damage either a direct one or due to a disease that affects the somatosensory nervous system. It is frequently characterized by hyperalgesia, abnormal pain sensitivity and can be appear together with allodynia, pain sensation following a neutral stimulus [90]. Here, we will briefly describe the role of CB2Rs on microglial activation in nerve injury models.

Several studies reported that CB2R agonists diminish pain sensitivity [91,92], but also reduce microgliosis and astrocytosis [93,94,95]. In agreement with that, CB2R overexpression decreased glial activation after sciatic nerve injury, whereas CB2R deletion caused enhanced pain accompanied by increased microgliosis [96].

Partial nerve ligation (PNL) causes chronic conditions with spontaneously reoccurring pain and hypoalgesia on the side of the injury (ipsilateral). Strikingly, CB2R knockout mice are one of the few animal models that develop also a contralateral pain, which suggests that CB2R signalling is necessary to restrict allodynia to the ipsilateral site [95,96,97]. Contralateral pain appeared more frequently after constitutive CB2R deletion as well as after CB2R deletion from myeloid cells, but did not appear after deletion of neuronal CB2R. Moreover, CB2R expression was induced upon PNL in dorsal root ganglia of the spinal cord in sciatic nerve microglia and macrophages, but not in neurons [97]. Thus, it seems that CB2Rs on microglia and macrophages in the spinal cord regulate pain responses.

On the contrary, another study suggested that rather neuronal and lymphoid and not myeloid CB2R is important for nociception after PNL [98]. This was shown by an increase in self-administration of a selective CB2R agonist, and an increased nociception after deletion of CB2R from neurons, but not from myeloid cells. Also, CB2-positive lymphocytes infiltrated the injury site and the depletion of CB2Rs from those cells recapitulated the phenotype observed in neuronal CB2R knockout mice. Strikingly, it was also suggested that CB2Rs might be transferred from invading lymphocytes to neurons, as a bone marrow transplant from CB2-GFP animals resulted in GFP-expression in dorsal root ganglion neurons. This expression was double in the PNL group in comparison to sham operated mice.

Taken together these results point to CB2R as an important modulator of neuropathic pain and suggest that the CB2R, not only on microglia, but also on other cell types, may play a role in neuromodulation.

#### 2.2.4. Traumatic Brain Injury (TBI)

In contrast to the chronic neuroinflammation that accompanies AD or HD, the TBI-induced neuroinflammatory process is acute. TBI is an injury of the brain, which is based on a sudden acceleration or deceleration within the cranium. It is induced by a biomechanical primary event, a trauma to the head, which is followed by secondary events, such as cell death, blood–brain-barrier disruption, excitotoxicity, mitochondrial disfunction, and neuroinflammation. These events subsequently result in neuronal disfunction and neuronal loss [99].

Earlier studies in mice demonstrated neuroprotective effects of the endocannabinoid 2-AG via CB1Rs in TBI [100]. As 2-AG also binds to CB2Rs, it is likely that CB2R activation also causes beneficial effects during TBI by modulating neuroinflammatory processes. Subsequently, enhanced CB2R expression was found in brain tissue [101,102] as well as on infiltrated myeloid cells in a mouse model of TBI where the damage is induced by controlled cortical impact [103]. Also, CB2R activation decreased the infiltration of macrophages and reduced the size of oedema after TBI and improved the motor function. In agreement with previous findings, CB2 receptor activation diminished the expression of pro-inflammatory mediators, while increased the expression of anti-inflammatory mediators. Although this study focusses on the role of infiltrating macrophages rather than on resident microglia, it suggests that CB2R activation mediates a shift from inflammatory M1 activation rather to an anti-inflammatory M2 activation [103]. Similar effects were observed in a study with mild TBI, where CB2R activation with the inverse agonist promoted an increase of M2 activation of microglia, simultaneously decreasing M1 activation. This resulted in a functional rescue of visualisation deficits caused by TBI [104].

Taken together, the findings from in vivo studies confirm the conclusion that CB2 receptors are indeed involved in inflammatory modulation and could be of great interest as therapeutical target. Especially as activation of CB2Rs, in contrast to CB1R activation, causes no psychoactive side-effects [105]. Although recent discovery of neuronal CB2Rs and its ability to alter neuronal transmission might challenge this view (see Section 1.2).

The summarized data especially from AD and TBI models support the hypothesis from in vitro findings stating that CB2R activation promotes a shift from M1 to M2 microglial state and thus promotes its anti-inflammatory action. However, still little is known how on a sub-cellular level does CB2R activity modulate microglial activation.

**Table 2:** Summary of CB2R-mediated effects on microglial activation/neuroinflammation in vivo. Molecular effects and behavioral phenotypes are either enhanced (↑), decreased (↓) or not altered (=).

Disease Model	Model	CB2R Activity Modification	Molecular Effects	Behavioral Phenotype	Reference
AD	**Pharmacological modification of CB2R activity**				
	APP/PS1 (APPswe/PS1dE9)	JWH-133	↓ Microglial activity ↓ IL-1β, IL-6, TNFα, IL-10 secretion ↓ Oxidative damage = Aβ plaque load	↑ V-maze	[65]
		Cannabidiol	= Aβ plaque load	↑ Novel object recognition ↑ Social recognition	[67]
		JWH-133	= Aβ plaque load ↓ Microglial activity ↓ IL6, TNFα, iNOS expression (region-dependent)	↑ Novel object recognition ↔ Spatial memory impairment (MWM)	[70]
	APP2576	WIN 55,212-2 JWH-133	↓ Microglial density (JWH-133) ↓ COX-2 ↓ IL-6, TNFα ↓ Aβ1-40 cortical levels	↑ Novel object recognition (JHW-133)	[69]
	AβPP23/PS45	HU-210	= APP processing and neuritic laque formation = Neurogenesis	= Spatial memory impairment (MWM) = Contextual fear conditioning	[66]
	**Genetic deletion of CB2R**				
	J20	*Cnr2*^tm1Dgen^/J	↑ Plaque-associated microglia ↑ soluble Aβ_42_ ↑ Aβ plaque load		[72]
	APP/PS1 (APPswe/PS1dE9)	*Cnr2*^tim1Zim^/J	↑ soluble Aβ_40_ = Aβ plaque load = Microglial activity	= Two-object recognition test	[71]
			↓ Microglial activity ↓ TNFα expression ↓ Aβ plaque load ↓ neuronal loss ↓ Infiltrating immune cells	↓ Spatial memory impairment (MWM)	[51,73]
HD	**Pharmacological modification of CB2R activity**				
	R6/2	GW405833	↓ Microglial activity ↓ Expression of IL-6	↓ Severity of motor symptoms	[86]
	***Genetic deletion of CB2R***				
	R6/2	*Cnr2*^tim1Zim^/J	↑ Microglial activity	↑ Severity of motor symptoms	[87]
	BACHD		↑ Expression of IL-6	↑ Onset and severity of motor symptoms	[86]
Neuropathic pain	**Pharmacological modification of CB2R activity**				
	Post-operative pain	JWH-015	↓ Microglial activity	↓ Paw incision–induced hypersen- sitivity	[93]
	Partial sciatic nerve ligation	β-caryophyllene JWH-133	↓ Microglial density (BCP)	↓ Pain response thermal sensitivity ↓ Pain response mechanical sensitivity	[95]
	Formalin Test	β-caryophyllene		↓ Pain response Formalin test	[95]
	Partial sciatic nerve ligation			↓ Pain response thermal sensitivity	[98]
TBI	**Pharmacological modification of CB2R activity**				
		GP1a	↓ Macrophage infiltration ↓ Oedema size ↑ Macrophage M2 polarization ↓ Expression of iNOS, TNFα, IL-6, IL-1β ↑ Expression of Arg1, IL-10	↓ Severity of motor symptoms	[103]
		Raloxifene	↓ M1/M2 microglia ratio ↓ Microglial density	↓ Severity of visual deficits	[104]
		SM-189	↓ M1/M2 microglia ratio ↓ Microglial density	↓ Severity of visual deficits	[106]

### 2.3. Intracellular Signalling Pathways

It is now widely accepted that the CB2R is able to manipulate microglial activity. However, the molecular mechanisms are not completely understood. CB2R, as a G-protein-coupled receptor, is coupled to Gα_i/o_ proteins, which inhibits adenylyl cyclase [107,108]. CB2R are also in a limited way coupled to Gα_q_ and calcium signaling [107,109]. Subsequently, Gα_i/o_, likely via Gβγ, stimulates cyclic adenosine monophosphate (cAMP) synthesis [110,111]. This in turn activates downstream protein kinase B (AKT) and extracellular-signal related kinase (ERK) signaling pathways that have been reported to be involved in microglia function (Figure 1B).

It was shown that phosphorylation of different mitogen-activated protein kinases (MAPK) (c-Jun N-terminal Kinase (JNK), ERK or p38) as well as nuclear factor ‘kappa-light-chain-enhancer’ of activated B-cells (NFkB) enhances the production of inflammatory mediators [112]. Among others, these pathways can mediate TNFα -production [113] and cell migration [114]. Moreover, CB2R signalling is involved in MAPK signalling (Figure 1B). An interaction of CB2Rs with the MAPK pathway was revealed in CB2R-overexpressing CHO-cells, as treatment with a CB2R agonist activated MAP kinases [115]. In contrast, CB2R activation with JWH-015 induced AKT and ERK, but not p38 MAPK signalling pathways in human monocytes [116]. Romero-Sandoval (2009) reported that CB2R activation with JWH-015 enhanced LPS-induced MPK1- and MPK3-expression, which in turn resulted in a reduced phosphorylation of ERK1/2 [117]. At the same time, CB2R activation induced an anti-inflammatory phenotype, as LPS-mediated TNFα expression and migration of primary microglia was reduced [117]. In addition, CB2R activation with JWH-015 was also able to inhibit IFNγ-mediated phosphorylation of JAK (Janus kinase) /STAT1 (signal transducer and activator of transcription). This blockade of JAK/STAT1 phosphorylation suppressed NO and TNFα-production in primary microglia [52].

Taken together, these data indicate that CB2Rs control microglial activity via MAPK pathways, which are also involved in downstream signalling pathways of the innate immune response. Nonetheless, a direct interaction of CB2Rs with receptors that take part in the immune response such as PRRs which are expressed by innate immune cells, is described only to a limited account. A variety of studies describe that CB2Rs are able to change LPS/TLR4 mediated microglial activation [54,55,118,119,120], but studies on a potential interaction of CB2/ECS and other TLRs are rare [121,122]. Thus, further investigations on the intracellular signalling of CB2Rs are needed to reveal other possible functions of the receptor.

### 2.4. CB2 Knockout Mouse Lines

To investigate the role of CB2Rs in inflammatory processes, both in vitro and in vivo studies used not only a pharmacological modulation of the receptor activity, but also genetic mouse models. Up to date there are two constitutive CB2R knockout strains: The Zimmer strain (*Cnr2*^tim1Zim^, [23]) and Deltagen strain (*Cnr2*^tm1Dgen^), in which the CB2R is deleted from all the cells in the body. Both knockout strains are on C57BL/6 background. The *Cnr2*^tim1Zim^ mouse line has a 131 amino acid deletion at the C-terminus [23,123], while the *Cnr2*^tm1Dgen^ strain targets the N-terminus with a 112 amino acid deletion (Deltagen).

Recently, the cre-lox technology enabled the generation of conditional knockout mouse lines, where CB2Rs are deleted in different cell types under the control of cell-type specific promoters. In those mouse lines, not only a part of the coding sequence, but the whole coding region of exon 3 and the upstream splicing site is flanked by loxP sites (*Cnr2*^fl/f^). When crossed to a cre-expressing mouse lines, site-specific recombination by Cre recombinase results leads to a complete deletion of CB2R in the respective cell/tissue.

As far as we know two groups independently generated *Cnr2*^fl/fl^ animals [21,124]. Up to date *Cnr2*^fl/fl^ were further crossed with three different cre-expressing mouse lines: Syn1-cre (B6.Cg-Tg(*Syn1-cre*)671Jxm/J) for neuron-specific deletion [21,97,98], DAT-cre for a specific deletion in midbrain dopaminergic neurons [124] and LysM-cre (B6.129P2-*Lyz2* tm1(cre)Ifo/J) animals for CB2 deletion in myeloid cells [97,98].

In the near future, crossing *Cnr2*^fl/fl^ with CX3CR1-cre mice would result in a microglia-specific deletion of CB2R. This mouse line could become a powerful tool to further study the role of microglial CB2R. Furthermore, using inducible Cre lines in which Cre recombination can be induced later on in life would aid in dissecting the role of CB2R in developing and adult brain.

Moreover, the generation of complete constitutive CB2R knockout animals using *Cnr2*^fl/fl^ mice might also explain some discrepancies from the comparison of *Cnr2*^tim1Zim^ and *Cnr2*^tm1Dgen^ results. It should be noted that both *Cnr2*^tim1Zim^ and *Cnr2*^tm1Dgen^ strains have been extensively used in many different studies, but surprisingly, not always giving the same results. It was speculated that since the deletion in *Cnr2*^tim1Zim^ does not include the promoter region, a truncated version of mRNA or/and protein can still be present in these mice and have some functional relevance [125], possibly acting as a dominant negative version of the receptor. In this case truncated receptor would interfere with primary functions of the receptor. However, previous studies did not detect any functional protein [23]. Furthermore, neuroinflammation was affected in both lines after the CB2R deletion. Surprisingly, CB2 deletion caused opposite effects depending on the mouse strain or of different magnitude as described above in context of diseases.

Some laboratories also studied constitutive CB2^−/−^ mice crossed to mice with other genetic backgrounds such as the outbred CD1 [126,127,128], FVB/NJ [86], or Biozzi ABH [125] strains. When interpreting the data from these studies, one should keep in mind that it is not known to what extent the genetic background can affect disease susceptibility.

Thus, CB2^−/−^ mice are a great tool to study the function of CB2 receptors as it also enables to study potential effects of CB2R-mediated inflammatory changes on behaviour. However, due to differences in experimental designs and strains used, the obtained data should be interpreted with caution. In the future, the generation of total constitutive knockouts deleting the whole gene as well as cell-specific ones could shed a light and solve some of the discrepancies.

### 2.5. CB2 Receptors on Microglia Influence Behaviour

#### Neuroinflammation and Cognition

Many studies suggest a direct link between neuroinflammation and cognitive performance. As CB2Rs play an anti-inflammatory role and is generally upregulated during a neuroinflammation, it was hypothesized that manipulating CB2R influences cognitive deficits, which frequently accompany neuroinflammatory processes.

Indeed, it was shown that CB2R activation can decrease cognitive dysfunctions in the mouse model of orthopaedic surgery [129]. In line with this finding, treating rats suffering from vascular dementia with HPβCD/BCP, a CB2R agonist, upregulated CB2R expression in the hippocampus and improved long-term spatial memory [130]. Moreover, as mentioned above, many different reports investigated the CB2 receptor in the context of AD and showed that the modulation of this receptor can influence learning and memory [51,72,73,131].

In conclusion, it seems like CB2R regulation during inflammation can affect cognition.

Further studies tried to dissect the role of neuronal and microglial CB2Rs under physiological conditions. CB2Rs regulates many different behaviours, from feeding [132,133,134] to addiction [35,47,124]. Among others, studies done in CB2^−/−^ mice hinted to an important role of CB2Rs in learning in memory, since it was shown that a constitutive CB2R deletion disrupts memory consolidation of an aversive memory accompanied by a decrease in synapse numbers [135]. Moreover, systemic administration of AM630, a CB2R antagonist impaired aversive memory, while administration of JWH133, while CB2R agonist had an opposite effect [135]. In agreement with that, CB2^−/−^ mice have an impairment in hippocampus-dependent, long-term contextual fear memory, but not in hippocampus-independent, cued fear memory [136]. These findings are consistent with a decreased synaptic density in the hippocampus that suggests decreased cognitive abilities [135,136]. Interestingly, CB2R deletion seems to enhance the working memory of the mice, as shown by an increased percentage of alternations in the Y-maze task [136]. Moreover, it was shown that 2-AG enhances memory consolidation via the activation of CB2Rs, and not CB1Rs, through the mammalian target of rapamycin (mTOR) pathway modulation [137].

To determine if neuronal and microglial CB2Rs play distinctive roles in the murine dorsal hippocampus, the CRISPR-Cas9 genome-editing technique and Cre-dependent overexpression combined with viral injections and transgenic mice were used to either knockout or overexpress the CB2R in specific cell types [138]. Three different cell types were targeted: glutamatergic, pyramidal neurons (using Camk2a promoter), GABAergic neurons (using Gad2 promoter), and microglia (using CX3CR1 promoter). Manipulation of CB2Rs on GABAergic neurons did not change any of the investigated behaviours, while CB2R deletion from pyramidal neurons enhanced working memory, as described before for constitutive KOs [136]. Contextual fear memory was enhanced after overexpression of microglial CB2Rs. Consistent with that, deletion of microglial CB2Rs decreased contextual fear memory. Pain sensitivity remained unchanged in these mice, indicated by stable foot shock thresholds. Moreover, a novel object recognition test showed an increased long-term memory after microglial CB2R deletion. The authors emphasized that their findings further support the expression of a functional CB2Rs in neurons and microglia. Since mice were not treated with any pro-inflammatory stimuli, the results rather reflect the role of CB2R in resting microglia, so under physiological conditions. Alternatively, it is also possible that stereotaxic intracranial injection could cause local inflammation that in turn would lead to the activation of microglia, but no sign of activation was found.

Altogether the studies showed that not only neuronal, but also microglial CB2Rs in the hippocampus play a role in learning and memory and that CB2Rs differently affect distinctive types of memory.

Considering the profound effects of cannabinoids on addiction-related behaviours, which are commonly thought to be mediated by CB1Rs, it was of great interest to determine if CB2R signalling also modulates the physiological and behavioural responses produced by drugs of abuse. Indeed, nicotine-induced conditioned place preference was completely absent in mice lacking CB2Rs and blocked by AM630 or SR144528 in wild type animals [126,139]. Cooperative effects of CB2R and nicotine receptors were suggested by the fact that the CB2R agonist O-1966 produced a conditioned preference when administered with a sub-threshold dose of nicotine [139]. Operant intravenous self-administration of nicotine was also reduced by the genetic deletion or pharmacological blockade of CB2Rs. These findings strongly indicate an involvement of CB2Rs in the reinforcing and rewarding effects of nicotine. Whether these receptors also contribute to the manifestation of nicotine physical dependence is unclear, as knockout studies produced conflicting results on mecamylamine-precipitated nicotine withdrawal responses [126,139].

Another drug of abuse for which CB2Rsignalling has been studied in some detail is cocaine. CB2R knockout mice showed a higher locomotor activity after injection of a single dose of cocaine (15 mg/kg) than wild type animals, indicating that the sensitivity to the locomotor effects of cocaine was increased in the absence of CB2Rs [127]. CB2R knockout mice displayed a normal cocaine conditioned place preference (CPP) and self-administration, but CB2R activation by the agonist O-1966 blocked cocaine CPP. These findings indicate that CB2R signalling has very different, even opposite effects on nicotine and cocaine reward. The results were further supported by the demonstration that systemic administration of the CB2R agonists JWH133 or GW405833 also attenuated cocaine self-administration, presumably by lowering the rewarding and/or motivational strength of cocaine [140]. It is conceivable that the systemic effects of CB2R agonists/antagonists, or the global deletion of CB2Rs, engage hitherto unknown peripheral mechanisms, rather than modulating cocaine and nicotine reward directly through neuronal actions. However, bilateral injection of JWH133 directly into the NAc, or intranasal microinjection of JWH133, which is thought to deliver the compound directly into the brain via the olfactory pathway, also inhibited cocaine self-administration, thus arguing against a peripheral mechanism. The specific involvement of CB2Rs in these experiments was supported by blockade of the effects by CB2R antagonists and the absence of effects in CB2R knockout mice [46,140]. A recent study demonstrated that CB2R activation blocked the acquisition and expression of both sensitization and CPP in cocaine-treated mice. This effect was opposed by administration of a CB2R antagonist. Furthermore, CB2R activation prevented neuronal activation in the hippocampus of CPP-exposed mice [141]. It should be noted that studies in rats produced results that were not entirely congruent with the findings in mice. Thus, the CB2R-selective agonist AM1241 and the antagonist AM630 failed to modify nicotine self-administration under fixed or progressive ratio schedules [142]. The CB2R antagonist SR144528 had also no effect on cocaine self-administration. Nevertheless, it reduced cocaine-induced (but not cue-induced) reinstatement of cocaine-seeking behaviour [143].

Taken together all the studies point into a key role of microglial CB2R in modulation of microglia function and neuroinflammation. Due to its role in modulating memory one could speculate if it modulates neuronal communication.

## 3. Open Questions and Controversies

### CB2Rs in Neuron-Microglia Interaction

Elements of the ECS are expressed by different cell types in the brain including neurons, microglia (see Section 1.2) and astrocytes [144]. Microglia and neurons do not only express CB2Rs, but also enzymes degrading and synthesizing two most abundant cannabinoids AEA and 2-AG [145,146,147] (Figure 1). This naturally raises the question, if the ECS plays a role in bidirectional neuron–microglia communication.

Control of microglia is crucial for maintaining homeostasis due to their defensive functions. Lack of an efficient, ongoing bidirectional communication might trigger microglia to switch to an activated phenotype and cause uncontrollable damage [148]. Nowadays many possible communication routs are still under investigation, but generally neurons are believed to be the ones controlling microglia and suppressing their offensive functions [149]. One possible mechanism through which such a control takes place is via a direct interaction of surface proteins [150,151,152]. An example of such a protein is the neuronally expressed chemokine fractalkine and its receptor CX3CR1, which is present on microglia [151]. Neuronal loss during neurodegeneration and consequent loss of these interacting surface proteins activates microglia, thus CX3CR1 is believed to be neuroprotective in neurodegenerative diseases [151]. We are not aware of any studies that directly investigated CB2R-mediated neuron–microglia interaction, but we have previously shown that CB1Rs on GABAergic neurons play a role in neuron–microglia communication via fractalkine/CX3CR1 [153]. Moreover, AEA, and possibly 2-AG as well, can be secreted via microglial extracellular membrane vesicles and by activating CB1R inhibit synaptic transmission of GABAergic neurons [154] (Figure 2). It is plausible that ECs released by microglia can also affect glutamatergic neurons, as this cell type also express CB1R, although in smaller amounts [155,156].

Another described mechanism of neuron-glia communication is through adenosine triphosphate (ATP) and purinergic receptors. Neuronal death due to neurodegeneration is accompanied by an excessive release of glutamate and ATP. ATP in turn can activate microglial purinergic ionotropic (P2X4, P2X7) and metabotropic (P2Y6, P2Y12) receptors. Glutamate, on the other hand can activate microglia via microglial N-methyl-D-aspartate (NMDA) channels, which in turn causes neuronal cell death [157]. It has been shown that microglia itself can also release ATP, raising a possibility of bidirectional neuron-glia communication using this compound. Interestingly, the release of shed microvesicles from the microglia surface can be triggered via activation of P2X7 by ATP [158] and a study showed that ATP-induced 2-AG production in microglia happens via P2X7 receptor activation [15,16].

The possible involvement of the ECS in neuro-glia crosstalk was also discussed in the context of neuropathic pain [159] (see Section 2.2). Glial cells and neuron-glia communication were implicated in the development of pain sensation after neutral stimuli (allodynia) and hypersensitivity (hyperalgesia). The exact mechanism is still unclear, but the most probable explanation is that activation of CB2Rs shifts microglia from M2 to M1 and thus decreases inflammation. One other possible communication route involves another chemokine—CCL2. CCL2 is synthesized de novo, released after peripheral nerve injury and activates cc-chemokine receptor 2 (CCR2) located on microglia [160,161]. Interestingly, CCL2 release is not increased after LPS/IFNγ stimulation in neonatal microglia of CB2^−/−^ mice [51] raising the possibility of a crosstalk between ECS- and CCL2-mediated neuron–microglial communication.

Another line of evidence that the ECS serves as a medium for microglia-neuron communication comes from studies investigating the role of the ECS in microglial regulation. A study speculated that microglial 2-AG in vitro regulates proliferation and migration in an autocrine fashion, so by binding to the microglial CB2R [16,145]. Moreover, it was shown that 2-AG takes part in shifting microglia from pro to anti-inflammatory phenotype (see Section 2.1) and plays a role in the proliferation and recruitment of microglia to migrate to the inflamed site [162]. AEA, being a potent CB2R agonist, could potentially also activate CB2Rs and contribute to its function.

On the other hand, similarly to AEA, microglial 2-AG could bind to the neuronal CB1R/CB2R regulating neuronal activity (Figure 1). Vice versa, we cannot exclude that the ECs produced by neurons could activate microglial CB2 receptor and shift the microglial state to the anti-inflammatory (M2) type as a neuroprotective mechanism.

Furthermore, ECs are synthesized on demand and CB2R expression is highly dynamic. CB2R is upregulated in neuroinflammation, but also in response to a behavioral context [163]. This makes the ECS a great candidate to play a role in neuron–microglia communication especially during an assault, when quick and precise reactions are needed. Of course, this does not exclude the possibility that it has a similar function under physiological conditions as expression of CB2Rs on microglia has also been shown in healthy brains (see Section 1.2).

**Figure 3:**
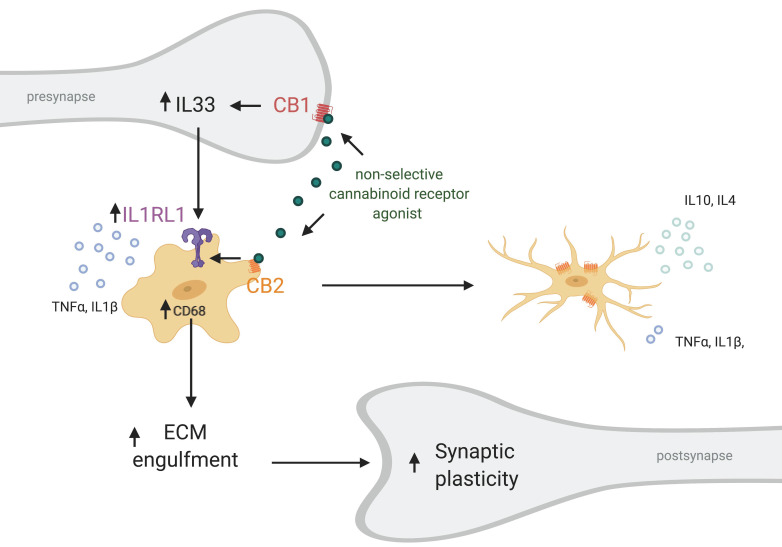
Putative role of CB2 in neuron-microglia communication through IL33–IL1RL1. Non-selective cannabinoid receptor agonist activates both CB1R on neurons and CB2R on microglia increasing the expression of IL-33 and IL1RL1, respectively. On one hand this leads to a shift from pro- to anti-inflammatory activation state (M1 to M2) of microglia, decrease in secretion of pro-inflammatory cytokines like TNFα and IL1β and increase in anti-inflammatory cytokines secretion including IL-10 and IL-4. Simultaneously, IL1R1 activation increases phagocytic capabilities of microglia via CD68 regulation and subsequently engulfment of extracellular matrix components by microglia. This in turn results is increased synaptic plasticity. Small arrow (↑) indicates an increase. Created with BioRender.com.

Recently, a novel neuron–microglia interaction was described that is involved in dendritic spine remodeling and memory consolidation [7]. In contrast to the traditional view considering neurons as the ones suppressing microglia activity, it was shown that neuronal release of IL33 can increase microglial phagocytic activity. IL-33 is upregulated and released by neurons upon experience—for example during learning or when surrounded by an enriched environment (Figure 3). It activates microglial IL1R1 which belongs to the TLR protein family (also known as ST2) that causes and upregulation of CD68. This in turn increases the phagocytic activity of microglia, in particular that of extracellular matrix components. Subsequently, the freed-up space enables for dendritic spines to remodel and even increase in numbers. Moreover, a recent investigation hinted that the ECS plays a role in IL33-IL1RL1 signaling. Treatment with WIN 55,212-2, non-selective cannabinoid receptor agonist, after carbon monoxide (CO) poisoning elevated protein levels of IL33 and IL1RL1 [164] (Figure 3). The treatment decreased cognitive deficits after CO poisoning, reduced neuronal loss and microglia density in the hippocampus. Furthermore, it reduced the levels of pro-inflammatory cytokines and shifted microglia from the M1 to M2 phenotype. Antagonizing CB1R with AM251 decreased IL33 levels, but did not later IL1RL1. On the contrary, the CB2 selective agonist AM630 decreased IL1RL1 levels, while not effecting IL33. Taken together this data suggests that CB2R interacts with the IL1RL1 signaling pathway and CB2R modulation alone can affect IL1RL1 levels hinting that CB2R plays a role in neuron–microglia communication. Unfortunately, the study did not include a control in which WIN 55,212-2 was administered to vehicle-treated individuals. It would have indicated if this mechanism is only relevant during inflammation or also under physiological conditions. Supporting these findings, it has been shown that constitutive CB2R deletion decreases dendritic spine density and the number of synapses [135,136], while chronic CB2R activation using JWH133 increases spine density [165].

Certainly, all those possibilities are very intriguing and could set the ECS as a key player in neuron–microglia communication, but further studies are needed to determine if and what role ECS plays in those interactions.

## 4. Conclusions

Ongoing studies are trying to pinpoint whenever microglia activation may be a cause of some pathologies or just associates with them. Either way, manipulating microglia activity seems to be a reasonable target for the treatment of many diseases accompanied by neuroinflammation, where neuron–microglia communication is frequently disturbed. The key seems to be not to inhibit microglia entirely, but to try to shift them from pro- to anti-inflammatory phenotype. Here, ECS and especially CB2R are brought to light as great candidates for targeted therapies, since CB2R activation switches microglia to an anti-inflammatory, pro-healing state and promotes migration to the site of the injury.

Unfortunately, until now knowledge about microglial CB2R is mostly based on in vitro studies. In vivo, CB2R is studied almost exclusively in the context of pathologies. Although overall in vivo studies also detected CB2R-related changes in neuroinflammation. However, to clearly state that the observed effects are due to the microglial CB2R usage of mouse models where CB2R is specifically deleted on microglia is crucial. Furthermore, inducible knockout models could also solve some discrepancies between results from knockout lines and pharmacological investigations.

Also, following investigations looking into molecular mechanisms of microglial CB2R action and its involvement in neuron–microglia communication will bring us closer to understanding its many functions and ways to manipulate it to treat a variety of diseases including neurodegenerative diseases.
